# Social support, psychological capital, multidimensional job burnout, and turnover intention of primary medical staff: a path analysis drawing on conservation of resources theory

**DOI:** 10.1186/s12960-024-00915-y

**Published:** 2024-06-19

**Authors:** Guimei Chen, Jing Wang, Qian Huang, Lingzhi Sang, Jing Yan, Ren Chen, Jing Cheng, Li Wang, Dongmei Zhang, Hong Ding

**Affiliations:** 1 School of Health Service Management, Anhui Medical University, 81 Meishan Road, Hefei, 230032 China; 2 Hospital Management Institute, Anhui Medical University, 81 Meishan Road, Hefei, 230032 China

**Keywords:** Job burnout, Social support, Psychological capital, Turnover intention, Conservation of resources theory, Primary medical staff

## Abstract

**Background:**

Job burnout is a prevalent and emerging challenge in the primary medical system, causing mass turnover, especially of primary medical staff. Little attention has been paid to the different dimensions of job burnout (emotional exhaustion, personality disintegration, and reduced sense of achievement), which may hinder efforts to tackle high turnover intention among primary medical staff. From the perspective of conservation of resources theory, social support and psychological capital are basic resources with potential to diminish job burnout and thus lower turnover intention. However, there is insufficient research evidence on the relationships between social support, psychological capital, and the three dimensions of job burnout within the primary medical system.

**Objectives:**

Focusing on primary medical staff, this study conducts a path analysis to examine the correlations between two types of resources (social support and psychological capital) and the three dimensions of job burnout, and to test the impact of the latter on turnover intention. Based on the results, effective management strategies to improve the work stability of primary medical staff are proposed.

**Methods:**

Multi-stage cluster random sampling was used to select participants in Anhui Province, China. Data were collected using a self-administered questionnaire containing measures of the main variables and demographic questions. In total, 1132 valid questionnaires were returned by primary medical staff. Structural equation modeling was used for path analysis of the data.

**Results:**

Social support was negatively associated with emotional exhaustion (*β* = − 0.088, *P* = 0.020), personality disintegration (*β* = − 0.235, *P* < 0.001), and reduced sense of achievement (*β* = − 0.075, *P* = 0.040). Moreover, psychological capital was negatively associated with emotional exhaustion (*β* = − 0.079, *P* = 0.030), personality disintegration (*β* = − 0.156, *P* < 0.001), and reduced sense of achievement (*β* = − 0.432, *P* < 0.001). All three dimensions of job burnout positively affected turnover intention (emotional exhaustion: *β* = 0.246, *P* < 0.001; personality disintegration: *β* = 0.076, *P* = 0.040; reduced sense of achievement: *β* = 0.119, *P* = 0.001).

**Conclusions:**

The results highlight the importance of social support and psychological capital for diminishing the three dimensions of job burnout for primary medical staff and, in turn, lowering their turnover intention. Accordingly, to alleviate job burnout and improve staff retention, material and psychological supports from leaders, colleagues, family, relatives, and friends are essential, as are measures to improve the psychological energy of primary medical staff.

## Introduction

Primary healthcare personnel play an irreplaceable role in accessible basic medical and public health services, against the background of a huge population and rising demand for health services in China [1]. Since the beginning of healthcare reform in 2009, China has been working to strengthen the primary healthcare system and improve its ability as a gatekeeper for generalist clinical care and basic public health services [2, 3]. Over recent years, with increasing demand for primary healthcare services, the phenomenon of job burnout has become more common among primary medical staff. The work overload and excessive demands they face were intensified by the outbreak of the COVID-19 pandemic. This situation has triggered a problem of high turnover, thereby blocking the progress of healthcare reform [4, 5]. Staff shortages caused by job burnout among primary medical staff has become a global problem [6]. One study estimated that the global shortage of medical providers, such as nurses and midwives, was 7.2 million in 2013, and predicted a sharp increase to 12.9 million by 2035 [7].

Job burnout refers to the depletion of emotional and physical energy resulting from prolonged stress at work [8]. It is a multidimensional concept, comprising emotional exhaustion (a feeling of one’s mental resources being eroded), personality disintegration (a distant attitude toward work tasks and disharmony in workplace relationships), and reduced sense of achievement (a feeling that one cannot complete tasks efficiently and adequately) [9]. Conservation of resources (COR) theory explains job burnout as the depletion of an employee’s emotional, cognitive, and physical energy resources [10]. It also posits that individuals attempt to offset the loss of resources through a defensive tendency or behavior [11]. According to studies of healthcare professionals, turnover intention is one outcome of job burnout [12, 13]. Turnover intention is an individual’s deliberate desire to quit their current job within a certain time period, and is a strong precursor of departure [7]. Although many healthcare studies have explored the job burnout–turnover intention relationship, few have specifically analyzed the roles of different dimensions of job burnout, especially in the literature on primary medical staff. In addition, previous research has found that the three dimensions of job burnout have different correlations with the same variable, such as psychological capital [14, 15]. Therefore, to enhance understanding of how to alleviate primary medical staff’s job burnout and, thereby, reduce their turnover intention, this study analyzes each individual dimension of job burnout.

Two important factors influencing job burnout are social support and psychological capital [16, 17]. Social support can be provided by workmates, relatives, family, and friends, which is a social relationship reflecting a connection of external resources [18, 19]. As a valuable internal resource, psychological capital is a positive mental ability comprising four elements: resilience, optimism, hope, and self-efficacy [20]. COR theory contends that social support and psychological capital are both strategic resources that can hinder stressors: whereas social support concerns “who you know,” psychological capital concerns “who you are” or “who you are going to be” [21]. There is a need to further explore the effects of social support and psychological capital on the different dimensions of job burnout among primary medical staff.

**Fig. 1:**
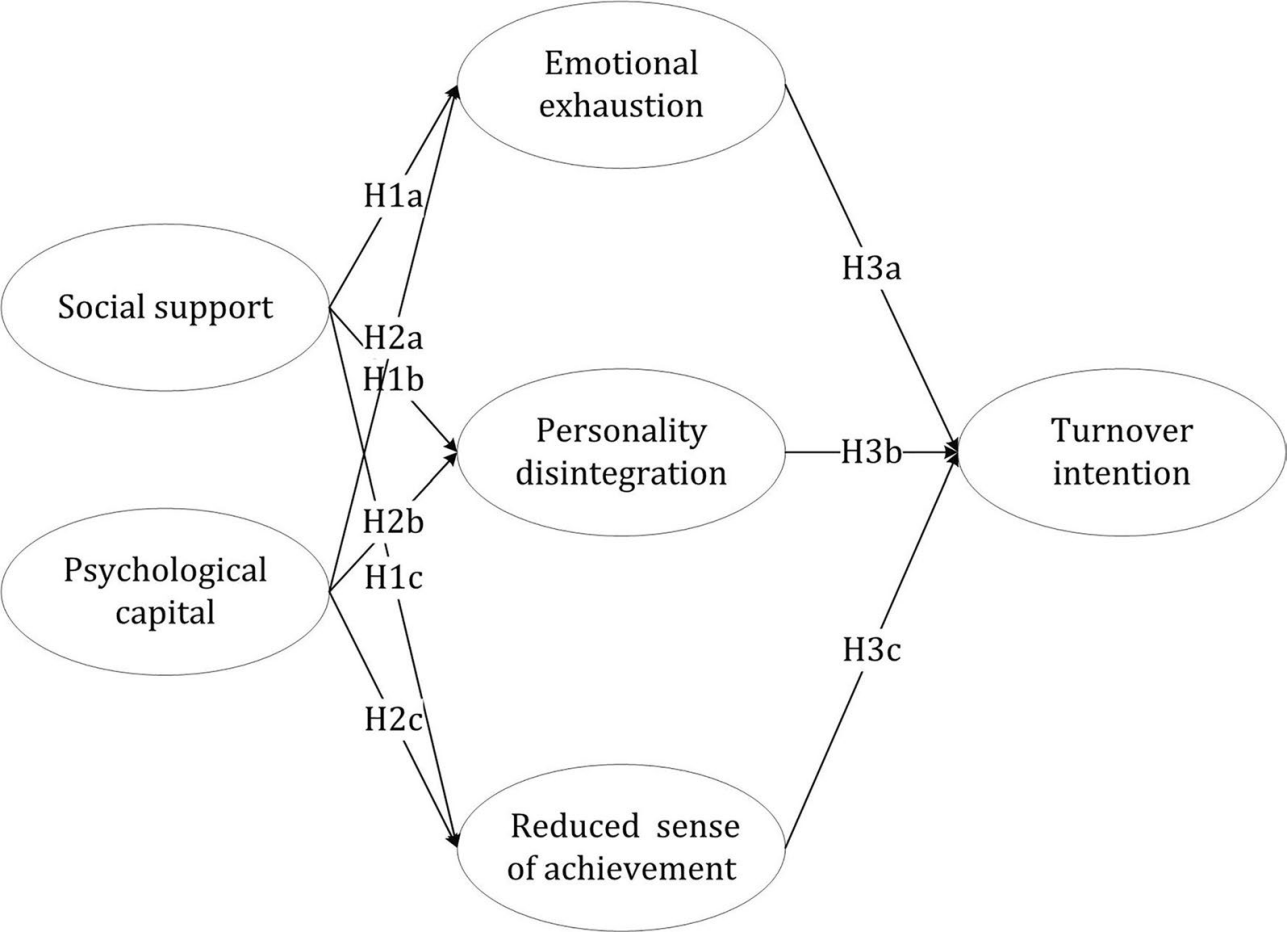
Study’s conceptual framework

Considering the importance of strengthening the primary medical system, and to address gaps in the literature, this study explores the antecedents of each dimension of job burnout and the respective associations between each dimension and turnover intention. Specifically, this study makes three main contributions. First, it applies COR theory to build a framework for the influence of social support and psychological capital on turnover intention, thus demonstrating the application of COR theory to healthcare professions. Second, this study reveals the specific effects of social capital and psychological capital on all three dimensions of job burnout. Finally, it enriches understanding of how each dimension of job burnout influences the turnover intention of primary medical staff (Fig. 1).

### Conservation of resources theory

COR theory offers a framework for how resources operate in individual and social systems [22], and is conducive to discerning the essence of stress as a universal phenomenon in every context related to people’s experience [23]. As Hobfoll proposed, the premise of COR theory is that individuals endeavor to acquire, preserve, and protect resources that they value [24]. These resources incorporate social support and positive personal energy [25]. Hobfoll further identified that stress occurs in reaction to situations, where resources are actually or potentially lost or where the investment of resources does not lead to more being obtained [26].

COR theory has been recognized as a suitable theoretical framework for understanding job burnout and consequently applied to many studies of this phenomenon [27]. Indeed, Lee and Ashford’s [28] meta-analysis of job burnout studies found that COR theory provides the best explanatory model. Based on COR theory, job burnout is a work-specific type of stress and represents a comprehensive response to the gradual depletion of an employee’s emotional, cognitive, and physical energy resources [10]. Furthermore, it has been noted that individuals with fewer resources at the early stage of job burnout tend to lose more material and mental resources if their endeavors to offset the depletion fail, and may then respond through defensive behaviors or behavioral intentions, such as turnover intention [29].

### Dimensions of job burnout

Job burnout is experienced by workers exposed to prolonged stress at work [30] and is a negative psychological state [31]. The term “burnout” was first introduced by American clinical psychologist Herbert Freudenberger in 1974, who considered staff burnout as a state of gradual physical and emotional depletion and loss of commitment and productivity among human service and healthcare volunteers [8].

At present, the most widely accepted definition of job burnout is that proposed by Maslach et al.—a psychological syndrome under chronic stressors and comprising the three dimensions of emotional exhaustion, personality disintegration, and reduced sense of achievement [1, 32]. Emotional exhaustion refers to an individual’s feeling of being emotionally drained, overextended, or fatigued from the job [33, 34]. Personality disintegration reflects a negative and callous attitude toward workmates and detached treatment of others in the workplace [34]. Reduced sense of achievement is a feeling of depleted confidence in one’s ability to work effectively or attain desired outcomes through one’s efforts [35].

Some studies have found differences in associations between the three dimensions of job burnout and related factors. For instance, Lizano and Barak [36] found that job demands predicted emotional exhaustion but not personality disintegration, and that the three dimensions differed in their impact on job satisfaction. Despite most of the literature indicating the rising prevalence of job burnout among primary medical staff, few studies have investigated the antecedents and consequences of the three dimensions of job burnout in this professional population [37, 38]. Hence, our study aims to fill this gap by drawing on COR theory.

### Social support and job burnout

The literature recognizes social support as an important externally available resource accessed via social relationships [34]. Taylor [39] regards social support as an experience of being cared for by others (e.g., family, relatives, friends, coworkers, and superiors) through the provision of psychological or practical aid, guidance, or other assistance. Accordingly, social support is also a job resource in the workplace [40].

Social support is likely to be linked to the three dimensions of job burnout. First, a person receiving social support may feel warm and valued by others, whereas one who lacks social support may feel isolated [41]. It has also been found that social support helps employees find meaning in their work and lives while dealing with job stress [42]. Hence, social support is likely to be associated with less emotional exhaustion. Second, social support reflects good social relationships and trust [43]. When an individual encountering stressful work receives social support from a coworker, they may feel empowered to develop coping strategies and thus create chances to discuss problems and share valuable information for solving work tasks together [34]. Thus, social support may reduce personality disintegration. Third, social support can bolster employees’ self-confidence, leading them to believe that ambiguous tasks provide opportunities to get ahead, rather than restrictions [28]. On this basis, social support is also likely to correlate with more personal accomplishment. Overall, our study hypothesizes that:

- H1a: Social support is negatively related to emotional exhaustion.
- H1b: Social support is negatively related to personality disintegration.
- H1c: Social support is negatively related to reduced sense of achievement.

## Psychological capital and job burnout

Applying the lens of positive organizational behavior, psychological capital was first described by Luthans et al. [20] as a person’s positive psychological capacities. Psychological capital is a composite construct, characterized by four mental resources: (1) strong belief in one’s ability to succeed and control outcomes when facing difficult challenges, termed self-efficacy [20]; (2) positive expectancy that motivates one to reach goals and avoid the negativity of unfavorable events, called optimism [21]; (3) self-motivation to create a realistic path toward achieving goals, termed hope [44]; and (4) recovering or adapting quickly following setbacks and failures, named resilience [45]. As a whole, psychological capital can be developed and helps to combat work stress [46].

Psychological capital is likely positively associated with lower levels of emotional exhaustion, personality disintegration, and reduced sense of achievement. More specifically, psychological capital is related to positive attitudes such as confidence, satisfaction, and a sense of empowerment [47, 48], which can help ease depression and mitigate loss of emotional capacity. Furthermore, higher levels of psychological capital are associated with greater ability to sustain working enthusiasm and bounce back when beset by adversity and then contribute to struggle to minimize risks and then increase chances of success [20, 49]. Moreover, individuals with higher psychological capital are likely to be more civil to coworkers [44], which may increase the chances of active collaboration on work tasks. Furthermore, in the healthcare field, one previous study found that psychological capital can decrease nurses’ job burnout [44].

Based on the literature, it is reasonable to expect that psychological capital is inversely related to the three dimensions of job burnout. Therefore, this study hypothesizes:

- H2a: Psychological capital is negatively related to emotional exhaustion.
- H2b: Psychological capital is negatively related to personality disintegration.
- H2c: Psychological capital is negatively related to reduced sense of achievement.

### Job burnout and turnover intention

Turnover intention can be described as the extent to which an individual considers quitting their current job and intends to seek employment elsewhere. It is also considered the final step in the decision-making process before an individual leaves their workplace [50, 51]. Previous research indicates that job burnout is a predictor of turnover intention [52].

Based on COR theory, individuals especially value their resources and thus seek to retain and protect them [24]. Where resources are potentially or actually lost, one may respond through defensive behaviors or behavioral intentions, such as intention to leave [53]. Given the poor living conditions and heavy workloads encountered by primary medical staff [24], the feeling of emotional exhaustion may also be accompanied by depersonalize the perception of work tasks or diminish feelings of accomplishment in the workplace [53], leading to less intention to remain in the same work environment [54]. Thus, this study hypothesizes:

- H3a: Emotional exhaustion is positively related to turnover intention.
- H3b: Personality disintegration is positively related to turnover intention.
- H3c: Reduced sense of achievement is positively related to turnover intention.

## Methods

### Ethics considerations

The data used in the present analysis was collected by the research team of Anhui Medical University, approved by the Ethical Committee of Anhui Medical University (review number AMUREC: 20170260).

### Sample and data collection

This study was conducted in Anhui Province, eastern China, which has an average economic development level. In 2020, there were 24 558 primary healthcare institutions in Anhui and a very large number of primary medical staff [55]. Anhui is a leading region of China’s comprehensive medical reform and provides strong grassroots services [56]. It was thus considered appropriate as a research area. To select participants for a cross-sectional survey, multi-stage cluster random sampling was carried out in Central Anhui, Northern Anhui, and Southern Anhui. One district and one county were selected for each of Central and Southern Anhui. Given the large population in Northern Anhui, we selected one district and two counties for that region. Potential respondents were selected from among all healthcare providers in work units (township hospitals, village clinics, community health service centers, community health service stations, and outpatient departments) in the selected regions. Data were collected through a self-administered questionnaire. Well-trained research investigators visited eligible work units over a 3-month period. All primary medical staff working on the day of the investigators’ visit were eligible to participate. Having the research investigators distribute and collect the questionnaires helped to ensure quality control.

The questionnaire comprised three parts. First, to ensure the validity of survey data, a brief introduction to the survey’s purpose was provided, and participants were assured that responses would be anonymous and participation was voluntary. The second part posed questions on basic demographic information, including gender, age, education level, occupation, and work units—all considered as control variables in this study. The third part included the main questions on the study variables. A total of 1300 questionnaires were returned. Following guidance from a prior study [57], responses with missing values or the same answer for every question were discarded as invalid. The final sample comprised 1132 participants, representing an effective response rate of 87.077%. Table 1 shows the general characteristics of the sample.

**Table 1:** Demographics of the sample (*N* = 1132)

Group	Frequency	Percentage (%)
Gender		
Male	515	45.495
Female	617	54.505
Age		
Under 31	176	15.548
31–40	361	31.890
41–50	495	43.728
51–60	91	8.039
61 and over	9	0.795
Education level		
Secondary school and below	488	43.110
Associate degree	443	39.134
Bachelor’s degree and above	201	17.756
Occupation		
Physicians	585	51.678
Pharmacists	71	6.272
Nurses	244	21.555
Medical managers	232	20.495
Work units		
Community health service centers	128	11.307
Community health service stations	47	4.152
Township hospitals	702	62.014
Village clinics	171	15.106
Outpatient departments	84	7.420
Regions		
North of Anhui	784	69.258
Middle of Anhui	212	18.728
South of Anhui	136	12.014

### Measurement development

The measurement scales were developed based on a detailed review of previous authoritative and publicly available research on job burnout, social support, psychological capital, and turnover intention. Some items were modified to fit the primary healthcare system in China. Appendix 1 shows information about the items of each construct and the values of standardized loadings.

Social support was measured with a simplified three-item scale originally devised by Dahlem et al. [58]. The social support scale was designed by Dahlem et al. to measure various supports provided by leaders, coworkers, family members, friends, and others [59]. A sample item is “Some people (leaders, relatives, colleagues) are there for me when I have a problem.” Responses to each item were given on a 7-point scale ranging from 1 (“entirely disagree”) to 7 (“entirely agree”). In this study, the scale’s Cronbach’s *α* was 0.799.

Psychological capital was measured by adapting the scale devised by Luthans et al. [20], which captures positive psychological capacities and included four items in this study. A sample item is “I always focus on the bright side of my work.” Responses to each item were given on a 7-point Likert scale ranging from 1 (“entirely disagree”) to 7 (“entirely agree”). The scale’s Cronbach’s *α* in this study was 0.786.

To measure job burnout, the Maslach Burnout Inventory (MBI) of Schutte et al. [60] was adapted. The MBI covers the three dimensions of job burnout, with every item answered on a 7-point Likert scale ranging from 1 (“entirely disagree”) to 7 (“entirely agree”). The first subscale measures emotional exhaustion, reflecting a loss of energy and chronic fatigue [61]. A sample item is “At the end of a day’s work, I feel very tired.” The subscale’s Cronbach’s *α* was 0.805. The second subscale measures personality disintegration, referring to a careless attitude toward work and reduced capacity to respond to coworkers [31]. A sample item is “I often blame the people I work with.” The Cronbach’s *α* of this subscale was 0.727. The third subscale measures reduced sense of achievement, reflecting negative feelings on successfully completing a task. All items were reversed scored, and a sample is “I can solve work problems effectively.” The subscale’s Cronbach’s *α* was 0.723.

Turnover intention was measured using items from Michael et al. [62]. A sample item is “I am considering whether to quit my present job.” Responses to each item were given on a 4-point Likert scale (1 = “never”; 2 = “seldom”; 3 = “once in a while”; 4 = “frequently”). In this study, the scale’s Cronbach’s *α* was 0.823.

The control variables were scored as follows: gender (1 = male, 2 = female); age (1 = under 31, 2 = 31–40, 3 = 41–50, 4 = 51–60, 5 = 61 and over); education level (1 = secondary school and below, 2 = associate degree, 3 = bachelor’s degree and above); and occupation (1 = physician, 2 = pharmacist, 3 = nurse, 4 = medical manager). Each was included to account for potential confounding variables based on previous studies [2].

## Results

### Measurement model test

SPSS statistical software and AMOS statistical software are the most commonly used tools for testing reliability and validity of the data and establishing the structural equation model [63]. In this study, SPSS 21.0 and AMOS 22.0 were used to test the measurement model.

Before carrying out path analysis, reliability and validity were tested. The reliability of each construct was examined by Cronbach’s alpha and composite reliability (CR) using SPSS 21.0. Table 2 shows Cronbach’s alpha values exceeded 0.70 for all variables [64], and all CR values were more than 0.80, thus exceeding the benchmark of 0.70 [65]. These findings confirm that the variables in the proposed research framework have good reliability. Each construct’s convergent validity was assessed by average variance extracted (AVE) and standardized loadings using SPSS 21.0. Table 2 also shows the standardized loadings of all variables’ items (0.634–0.898) exceeded the threshold of 0.60 [66], and the AVE of all variables (0.476–0.739) exceeded the critical value of 0.40 [67]. Discriminant validity is established if the square root of AVE of each variable exceeds that variable’s correlation coefficients with all other variables [67]. As shown along the diagonal in Table 3, for each variable, the square root of AVE exceeded the correlation coefficients with all other variables. These results confirm acceptable discriminant validity.

**Table 2:** Reliability and convergent validity

Construct	Cronbach’s alpha	Standardized loadings	CR	AVE
1. Social support (SS)	0.799	0.837–0.855	0.883	0.715
2. Psychological capital (PC)	0.786	0.738–0.835	0.863	0.612
3. Emotional exhaustion (EE)	0.805	0.814–0.869	0.885	0.720
4. Personality disintegration (PD)	0.727	0.768–0.832	0.849	0.652
5. Reduced sense of achievement (RSA)	0.723	0.634–0.736	0.819	0.476
6. Turnover intention (TI)	0.823	0.836–0.898	0.895	0.739

**Table 3:** Means, standard deviation, and correlations

	Mean	SD	1	2	3	4	5	6	7	8	9	10
1. Gender	1.550	0.498	–									
2. Age	2.466	0.877	− 0.269***	–								
3. Education	1.746	0.738	0.133***	− 0.302***	–							
4. Occupation	2.110	1.242	0.389**	− 0.211***	0.076*	–						
5. Social support (SS)	5.405	1.196	0.122***	− 0.025	0.044	0.108***	**0.846**					
6. Psychological capital (PC)	4.130	0.735	0.106**	0.006	− 0.035	0.096**	0.460***	**0.782**				
7. Emotional exhaustion (EE)	2.903	1.148	− 0.110***	0.019	− 0.008	− 0.111***	− 0.083**	− 0.109***	**0.849**			
8. Personality disintegration (PD)	1.903	1.010	− 0.221***	0.138***	− 0.173***	− 0.173***	− 0.235***	− 0.211***	0.311**	**0.807**		
9. Reduced sense of achievement (RSA)	2.423	0.880	− 0.080**	0.022	− 0.057	− 0.065*	− 0.192***	− 0.347***	− 0.178***	0.088**	**0.690**	
10. Turnover intention (TI)	2.051	0.892	− 0.126***	− 0.010	− 0.055	− 0.199***	− 0.230***	− 0.183***	0.240***	0.179***	0.058	**0.860**

*SS* social support, *PC* psychological capital, *EE* emotional exhaustion, *PD* personality disintegration, *RSA* reduced sense of achievement. *TI* turnover intention

SD means standard deviation. The diagonal elements in bold are the square roots of the average variance extracted (AVE), and the off-diagonal elements are the correlation between constructs

*P < 0.05, **P < 0.01, ***P < 0.001

Confirmatory factor analysis was performed using AMOS 22.0 to test the adequacy of multi-item scales in capturing their respective constructs. The following fit indexes were used: the ratio of *χ*^2^ to degrees of freedom (*χ*^2^/*df*), the comparative fit index (CFI), the Tucker–Lewis index (TLI), the standardized root mean square residual (SRMR), and the root mean square error of approximation (RMSEA) [68]. The results show that the measurement model has good fit and acceptable discriminant validity: *χ*^2^/*df* = 2.749 < 5.0, TLI = 0.958 > 0.90, CFI = 0.966 > 0.90, SRMR = 0.038 < 0.05, RMSEA = 0.039 < 0.08.

To test for potential multicollinearity, variance inflation factors (VIFs) were estimated using regression analyses, with turnover intention as the dependent variable and with social support, psychological capital, the three dimensions of job burnout, and control variables as independent variables. This test was performed using SPSS 21.0. The VIFs ranged from 1.104 to 1.443, and so were all below the threshold of 10 [69]. Hence, there was no serious multicollinearity problem in the dataset.

### Common method variance

Harman’s single-factor model was applied to examine common method bias in the data [70]. The results showed that the first factor explained only 21.30% of total variance, which is below the threshold of 50%, indicating that common method variance was not a serious problem. Moreover, a confirmatory factor analysis was conducted including an unmeasured latent common method factor (ULCMF) [71]. Table 4 reports that the multi-factor model was significantly better than the single-factor model (∆*χ*^2^ = 4087.026, ∆*df* = 15, *P* < 0.001) and not significantly different from the multi-factor model with a ULCMF (∆CFI = 0.000, ∆TLI = 0.001, ∆RMSEA = 0.001). These consistent results show that common method variance was minimal.

**Table 4:** Fit indices for the measurement models

Model	*χ*^2^	*df*	RMSEA	CFI	TLI	SRMR
Multi-factor model (SS, PC, EE, PD, RSA, TI)	376.676	137	0.039	0.966	0.958	0.038
Single-factor model (SS + PC + EE + PD + RSA + TI)	4463.702	152	0.158	0.392	0.316	0.141
Common method factor model (multi-factor model, ULCMF)	376.676	136	0.040	0.966	0.957	0.038

*ULCMF* an unmeasured latent common method factor

### Structural model test

**Fig. 2:**
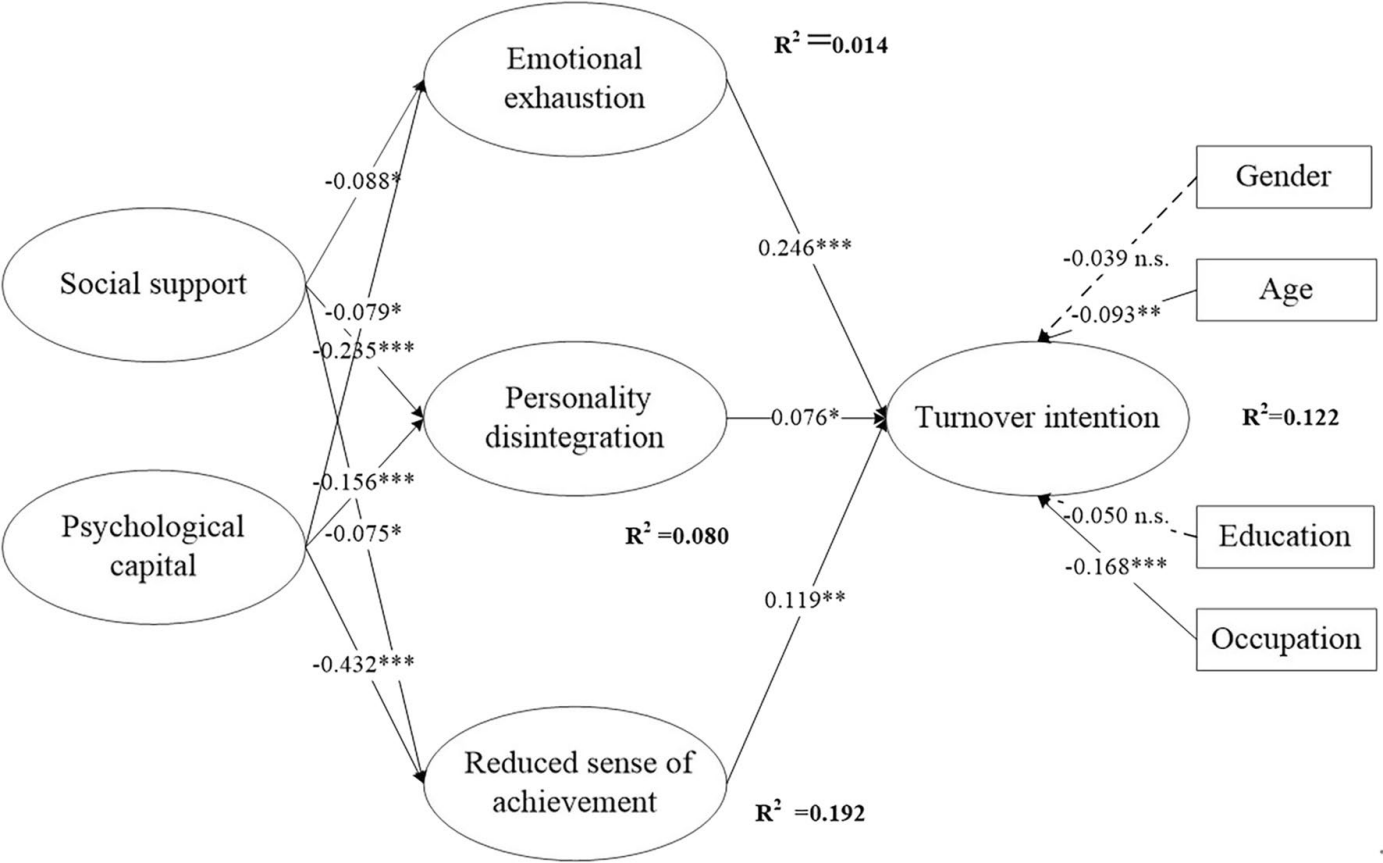
Structural equation model analysis of the research model

A good measurement model of latent variables is a prerequisite for causal analysis between latent variables and for building structural equation models. Our results of confirmatory factor analysis indicated that the discriminant validity of all variables was satisfactory, and the common method variance was not a serious problem. However, the results of confirmatory factor analysis do not explain structural relationships between variables [72]. Hence, the structural equation model was conducted. Figure 2 presents the results of the structural equation model. Model fit with the dataset was acceptable (*χ*^2^ = 1059.961; *df* = 215; *χ*^2^/*df* = 4.930 < 5.0; goodness-of-fit index (GFI) = 0.925 > 0.90; adjusted goodness-of-fit index (AGFI) = 0.904 > 0.90; root mean square error of approximation (RMSEA) = 0.059 < 0.08). The path analysis values are shown in Table 5. All the hypothesis results were consistent with the expected directions. First, social support was negatively associated with emotional exhaustion (*β* = − 0.088, *t* = − 2.387, *P* = 0.020), personality disintegration (*β* = − 0.235, *t* = − 6.000, *P* < 0.001), and reduced sense of achievement (*β* = − 0.075, *t* = − 2.024, *P* = 0.040), indicating that H1a, H1b, and H1c are supported. Second, psychological capital was also negatively correlated with emotional exhaustion (*β* = − 0.079, *t* = − 2.153, *P* = 0.030), personality disintegration (*β* = − 0.156, *t* = − 4.044, *P* < 0.001), and reduced sense of achievement (*β* = − 0.432, *t* = − 9.652, *P* < 0.001), indicating that H2a, H2b, and H2c are supported. Third, emotional exhaustion (*β* = 0.246, *t* = 6.843, *P* < 0.001), personality disintegration (*β* = 0.076, *t* = 2.114, *P* = 0.040), and reduced sense of achievement (*β* = 0.119, *t* = 3.245, *P* = 0.001) positively affected turnover intention. Thus, H3a, H3b, and H3c are supported.

**Table 5:** Path analysis coefficients of the structural model

Path	Standardized estimate	*P* value	S.E.	*t* value	Result
H1a: SS → EE	− 0.088*	0.020	0.032	− 2.387	Accept
H1b: SS → PD	− 0.235***	< 0.001	0.034	− 6.000	Accept
H1c: SS → RSA	− 0.075*	0.040	0.023	− 2.024	Accept
H2a: PC → EE	− 0.079*	0.030	0.055	− 2.153	Accept
H2b: PC → PD	− 0.156***	< 0.001	0.057	− 4.044	Accept
H2c: PC → RSA	− 0.432***	< 0.001	0.049	− 9.652	Accept
H3a: EE → TI	0.246***	< 0.001	0.028	6.843	Accept
H3b: PD → TI	0.076*	0.040	0.029	2.114	Accept
H3c: RSA → TI	0.119**	0.001	0.039	3.245	Accept
Gender → TI	− 0.039	0.260	0.050	− 1.131	Reject
Age → TI	− 0.093**	0.005	0.028	− 2.782	Accept
Education → TI	− 0.050	0.130	0.032	− 1.534	Reject
Occupation → TI	− 0.168***	< 0.001	0.020	− 4.930	Accept

S.E.: standard error of regression weight

*P < 0.05, **P < 0.01, ***P < 0.001

The results also illustrate that the age (*β* = − 0.093, *t* = − 2.782, *P* = 0.005) and occupation (*β* = − 0.168, *t* = − 4.930, *P* < 0.001) of primary medical staff were significantly related to turnover intention. Conversely, neither gender (*β* = − 0.039, *t* = − 1.131, *P* = 0.260) nor education level (*β* = − 0.050, *t* = − 1.534, *P* = 0.130) significantly influenced turnover intention.

## Discussion

### Theoretical implications

This study investigated two kinds of antecedents and one outcome of job burnout, uncovering the relationships among social support, psychological capital, the three dimensions of job burnout, and turnover intention in China’s primary medical staff. The major research findings are as follows. First, social support significantly negatively affected emotional exhaustion, personality disintegration, and reduced sense of achievement. These findings indicate that social support, as a basic resource [73], plays an important role in alleviating physical and mental attrition in the workplace. The results are also similar to prior findings of a negative relationship between social support and job burnout in healthcare professionals [74]. However, earlier studies, especially of primary medical staff, considered job burnout as an overall variable and did not explore its different dimensions. Our study offers empirical evidence that social support from family, relatives, friends, leaders, and colleagues can reduce psychological and emotional depletion, loss of commitment, and reduced sense of achievement in primary medical staff facing an excessive workload.

Second, psychological capital was found to be significantly negatively associated with all three dimensions of job burnout. Prior studies have also reported that psychological capital can negatively predict job burnout in healthcare professionals, for instance under the pressure of COVID-19 [75], but without analyzing the different dimensions of job burnout. Interestingly, our results are not consistent with a Spanish study [15], in which psychological capital was found to be related to reduced sense of achievement but not emotional exhaustion or personality disintegration. The inconsistency may be explained by differences in the research design: specifically Meseguer de Pedro et al. [15] conducted a long-term study (two research periods), so other factors could have led to the insignificant relationships of psychological capital with emotional exhaustion and personality disintegration. Moreover, the authors suggested that the symptoms of emotional exhaustion and personality disintegration may have been too overwhelming for individuals with a low level of psychological resources [15]. In our study, by contrast, psychological capital equipped primary medical staff with the capacity to deal with emotional exhaustion, personality disintegration, and reduced sense of achievement in the workplace.

Third, as hypothesized, all three dimensions of job burnout were found to be significantly positively related to turnover intention. These results reinforce many prior findings that job burnout overall aggravates turnover intention in healthcare professionals [37]. Furthermore, the findings are also in line with a previous study [46] reporting significantly positive associations between the three dimensions of job burnout and turnover intention among healthcare providers in township health centers. Our study shows that those relationships hold in a broader range of primary healthcare units, including not only township hospitals but also village clinics, community health service centers, community health service stations, and outpatient departments. Therefore, our results confirm the important influence of all three dimensions of job burnout on the turnover intention of healthcare professionals.

Overall, this study makes three main theoretical contributions. First, it expands the application of COR theory in the field of primary health care, introducing a holistic framework for the impacts of different resources (social support and psychological capital) on job burnout and turnover intention. Second, this study analyzes antecedents of the three dimensions of job burnout and how each dimension influences turnover intention, thereby expanding the literature on turnover intention in primary medical staff. Finally, the results confirm that all three dimensions of job burnout could lead to turnover intention with data from five types of primary healthcare institution, thereby expanding prior findings from the study of township health centers only.

### Practical implications

At present, China’s primary medical staff face heavy workloads and high work intensity, leading many to choose to leave their jobs [6]. Meanwhile, the shortage of medical staff makes workloads even heavier for remaining staff [76]. Although the shortage of medical staff cannot realistically be solved quickly, it is necessary to take actions to lower the high level of turnover intention in primary healthcare institutions. From the perspective of resource acquisition and utilization, we can alleviate job burnout using existing physical and mental resources in the primary healthcare system [46].

The results of our study have some important implications for governments, primary healthcare institutions, and others seeking to address job burnout and high turnover intention in primary medical staff. Our findings reinforce that social support can ease job burnout. In previous studies, social support could not alleviate the influence of job stress on feelings of burden but did alleviate burnout [77]. Moreover, primary medical staff who received adequate social support were found to be better able, both physically and mentally, to cope with job burnout [78]. Thus, people from all walks of life, especially leaders and colleagues in primary healthcare institutions, family, relatives, and friends should take an active interest in the working conditions of jaded primary medical staff. For example, leaders and colleagues could share the benefit of their stress-handling skills and experience with primary medical staff facing high job burnout [79], while family and friends could listen, give advice, and relieve their distress and other harmful impacts of work stress [80]. Our findings also demonstrate that psychological capital represents a positive mental resource that could help to lower job burnout. Accordingly, governments and primary healthcare institutions should focus on improving the psychological capital of primary medical staff, for instance by organizing mental training and courses to raise confidence and resilience. The practices suggested above may relieve job burnout and thereby reduce turnover intention in primary medical staff.

### Limitations and future research

This study has some limitations, which future studies should aim to address. First, our data were gathered in one province of China, so the results may not be generalizable to other cultural contexts in different countries. Future research should collect and analyze data from diverse cultural contexts to further test the validity of our results. Second, this study’s cross-sectional design prevents identification of causal associations between variables. Future studies should adopt a longitudinal approach to investigate causation. Third, this study does not investigate the dimensions of social support and psychological capital; doing so could extend our research conclusions. In addition, we did not consider the working environment factors likely to contribute to job burnout in primary medical staff, such as sufficient staffing, interprofessional relationships, authentic leadership and workplace empowerment [78]. Future research could incorporate such factors. Finally, some economic supports such as retention incentives, housing and transportation support and family economic supports could be taken into account in the future studies to build a comprehensive framework influencing psychological capital, burnout and job turnover of primary medical staff.

## Conclusion

Drawing on COR theory, our study conducted a path analysis to examine the antecedents and consequences of different dimensions of job burnout among primary medical staff. The findings reveal that both social support and psychological capital were negatively related to emotional exhaustion, personality disintegration, and reduced sense of achievement. Moreover, all three dimensions of job burnout profoundly aggravated turnover intention. From the perspective of resource preservation and acquisition, it is necessary to give primary medical staff social support and opportunities to strengthen their psychological capital, thereby helping to reduce pressure in the workplace. Based on the findings, we propose several strategic suggestions for better developing medical and health services: in particular, it is essential to pay attention to the occupational mental health of primary medical staff by offering adequate material resources and mental health support from leaders, colleagues, family, relatives, and friends. In addition, more training activities should be provided to help primary medical staff build psychological energy and enhance their ability to resist pressure.

## Data Availability

Taken into account the anonymity of the participant and the datasets is being used in other unpublished studies, the datasets for this article are not publicly available. However, if it is reasonable requested, please contact the corresponding author to obtain our datasets.

## Appendix 1. Measurement scale

**Table:**

Construct	Item	Measurement	Standardized loading
1. Social support (SS) adapted from Dahlem et al. [58]	SS1	Some people (leaders, relatives, colleagues) are there for me when I have a problem.	0.844
	SS2	My family can help me concretely.	0.837
	SS3	My friends can really help me.	0.855
2. Psychological capital (PC) adapted from Luthans et al. [20]	PC1	In my line of work, I believe I can help set goals/objectives.	0.774
	PC2	I can think of many ways to achieve my current job goals.	0.835
	PC3	I usually take the pressure at work calmly.	0.779
	PC4	I always focus on the bright side of my work.	0.738
3. Emotional exhaustion (EE) adapted from Schutte et al. [60]	EE1	I'm very tired.	0.814
	EE2	I often feel exhausted.	0.861
	EE3	At the end of the day's work, I felt very tired.	0.869
4.Personality disintegration (PD) adapted from Schutte et al. [60]	PD1	The people I work with often complain about me.	0.768
	PD2	I often blame the people I work with.	0.832
	PD3	I often refuse requests from people I work with.	0.821
5.Reduced sense of achievement (RSA) adapted from Schutte et al. [60]	RSA1	I can solve work problems effectively. (Reversed item)	0.634
	RSA2	I can effectively influence others through my work. (Reversed item)	0.645
	RSA3	I can create a relaxed and lively working atmosphere. (Reversed item)	0.736
	RSA4	I'm very excited after solving the problem of my work object. (Reversed item)	0.727
	RSA5	I accomplished a lot of meaningful tasks. (Reversed item)	0.702
6.Turnover intention (TI) adapted from Michaels et al. [62]	TI1	I am considering whether to quit my present job.	0.898
	TI2	I'm wondering if I should look for other similar jobs.	0.844
	TI3	I'm thinking about looking for a different kind of job.	0.836

## Abbreviations

COR: Conservation of resource
CR: Composite reliability
AVE: Average variance extracted
CFI: Comparative fit index
AGFI: Adjusted goodness-of-fit index
TLI: Tacker–Lewis index
SRMR: Standardized root mean square residual
RMSEA: Root mean square error of approximation
VIFs: Variance inflation factors
ULCMF: Unmeasured latent common method factor
